# Prognostic role of urinary collecting system invasion in renal cell carcinoma: a systematic review and meta-analysis

**DOI:** 10.1038/srep21325

**Published:** 2016-02-18

**Authors:** Luyao Chen, Hongzhao Li, Liangyou Gu, Xin Ma, Xintao Li, Fan Zhang, Yu Gao, Yang Fan, Yu Zhang, Yongpeng Xie, Xu Zhang

**Affiliations:** 1 State Key Laboratory of Kidney Diseases, Department of Urology, Chinese PLA Medical School, Chinese PLA General Hospital, Beijing, People’s Republic of China; 2 Medical School, Nankai University, Tianjin, People’s Republic of China

## Abstract

The relationship between urinary collecting system invasion (UCSI) and oncological outcomes in renal cell carcinoma (RCC) patients has attracted extensive attention recent years. However, the reports were inconsistent and remain controversial. Thus, we performed a systematic literature search of PubMed, Embase, Web of Science and The Cochrane Library databases to identify relevant studies up to June 2015 and conducted a standard meta-analysis of survival outcomes. 17 studies containing 9012 RCC patients satisfied the inclusion criteria. Pooled HRs for overall survival (OS) and recurrence-free survival (RFS) were 1.45 (95% CI, 1.26–1.66, P < 0.001) and 2.27 (95% CI, 1.54–3.34, P < 0.001), respectively. Further subgroup analysis suggested that UCSI was significant associated with poor cancer-specific survival (CSS) in stage T1–T2 RCC (HR = 2.05, 95% CI: 1.43–2.96, P < 0.001) but not in stage T3–T4 tumors (HR = 1.08, 95% CI: 0.63–1.85, P = 0.771). Current evidence revealed that UCSI has a significant negative impact on OS and RFS in RCC patients and could be used to predict CSS especially in localized RCC. Thus, RCC patients with UCSI should be paid more attention by clinician and pathologist and require close follow up for their poor prognosis.

Renal cell carcinoma (RCC) is the third most common genitourinary tumor, which represents about 3% of all human cancers^1^. Epidemiological data demonstrate that the incidence of RCC has steadily increased in recent years^2^. The increase may be due to the widespread use of non-invasive imaging techniques, which allow the early detection of small renal masses^3^,^4^. With approximately 25–30% of patients found to have metastases at presentation and 20–30% of patients occurs relapse after surgical resection^5^, RCC patients should be closely watched and stratified to categories with different risk of recurrence, progression and survival. The stratification can significantly improve postoperative patients’ counseling, selection of individualized treatment, planning of appropriate follow-up schedules and the design of clinical randomized controlled trials. Currently the Tumor-Nodes-Metastasis (TNM) staging system remains the most widely accepted system for RCC classification, which describe the anatomic extent of RCC and relate it to the prognosis^6^. However, patients with comparable tumor characteristics can experience significantly different even opposed clinical outcomes. Thus, it is necessary for the staging system to be continuously assessed and updated as new research data are available.

Invasion of urinary collecting system (UCSI), which used to be included in the first edition of American Joint Committee on Cancer (AJCC) TNM staging system in 1978, is no longer considered as a criterion in the subsequent versions^7^. However, some recent studies have focused on the prognostic value of UCSI in RCC and suggested inconsistent and controversial results. Anderson *et al*.^8^ found that the presence of UCSI was independently associated with higher overall and disease-specific mortality in patients undergoing nephrectomy for locally invasive RCC. Brookman-Amissah *et al*.^9^ also demonstrated that collecting system invasion was independently associated with a significant decline in cancer-specific survival and was associated with simultaneous metastatic spread at the time of surgery and multilocular dissemination. On the other hand, Waalkes *et al*.^10^ and Schrader *et al*.^11^ suggested that UCSI was disqualified as individual prognostic factor for RCC and did not advocate the inclusion of UCSI into upcoming TNM staging systems.

Understanding the relationship between UCSI and RCC outcomes is very important for the prognostic models establishing. To derive a more precise evaluation of the prognostic significance of UCSI in RCC patients, we systematically review published relevant studies and carried out a meta-analysis by standard techniques.

## Results

### Study characteristics

**Figure 1:**
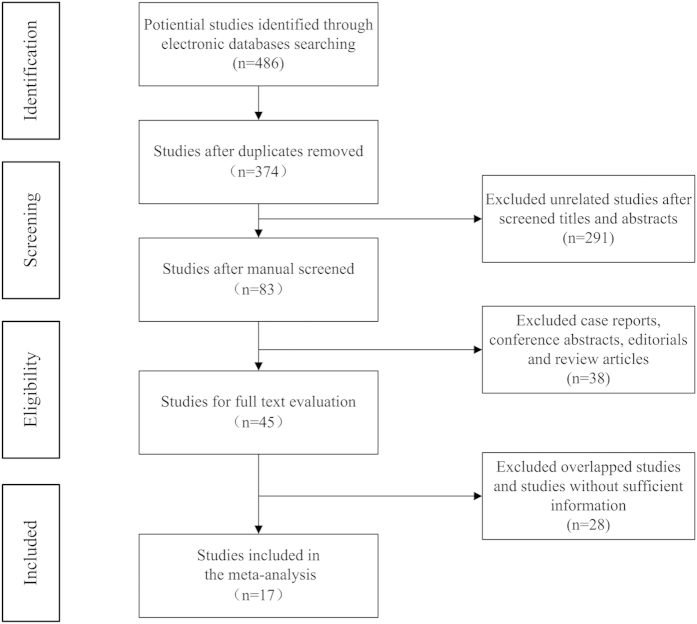
Flow chart of study selection.

A total of 486 potential relevant studies were retrieved from our initial literature search in the aforementioned databases. Using literature manager software (Endnote), 112 duplicated papers were excluded. After carefully screening titles and abstracts of identified records, 329 studies were excluded for reasons such as apparent irrelevant studies, case reports, conference abstracts, editorials and review articles. Of the remaining 45 studies selected for full text evaluation, 28 studies that belonged to duplicated publication, or failed to offer sufficient data (HRs with corresponding 95% CI) were excluded. Finally, 17 studies met our eligibility criteria and were included in the meta-analysis (Fig. 1). The characteristics and information of these included studies were shown in Table 1. The 17 studies contained 9012 RCC patients including 1008 UCSI, which were all diagnosed by histopathological methods. These RCC patients came from different countries (China, United State, Brazil, Korea, Italy, Egypt, Germany and France) with the duration of follow-up of more than 12 months. Of the eligible 17 studies, 12 studies^8^,^9^,^10^,^11^,^12^,^13^,^14^,^15^,^16^,^17^,^18^,^19^ containing 7006 patients were carried out to investigate the impact of UCSI on the CSS of RCC patients, 4 studies^8^,^18^,^20^,^21^ containing 2086 patients to investigate the OS and 4 studies^17^,^22^,^23^,^24^ containing 1157 patients reported the RFS, respectively. Assessment of quality scores by NOS demonstrated that the scores of included studies ranged from 7 to 9, which were considered adequate for the following meta-analysis.

### Meta-analysis

**Figure 2:**
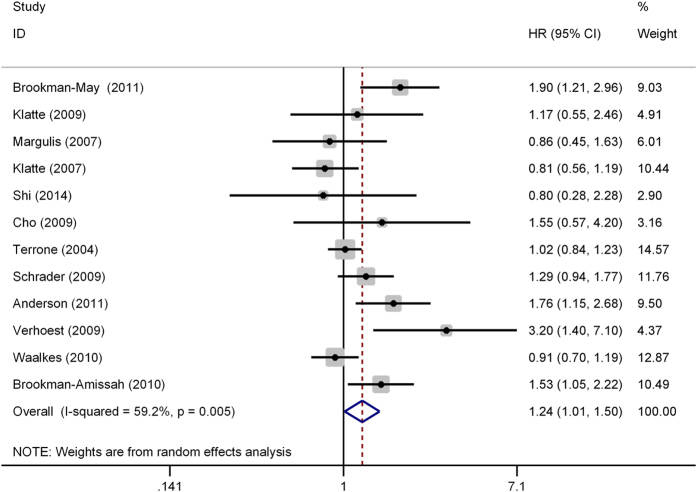
Forest plot of studies evaluating the association between urinary collecting system invasion and cancer-specific survival of renal cell carcinoma. HR = hazard ratio; CI = confidence interval.

Of the 12 studies that referred to CSS, there was apparent inter-study heterogeneity (*I*^*2*^ = 59.2%, P = 0.005). Thus, a random effect model was performed to calculate the pooled HR and corresponding 95% CI. As shown in Fig. 2, the combined HR of these studies revealed that UCSI was associated with poorer CSS in RCC patients (HR = 1.24, 95% CI: 1.01–1.50, P = 0.036). To explore the source of significant heterogeneity, meta-regression analysis and subgroup analysis were performed by patients ethnicity, study number, tumor stage and analysis style. The results showed that tumor stage might have significant association with the heterogeneity (P = 0.041), while other factors did not (Table 2). In addition, subgroup analysis showed that the combined HR estimate for CSS in Caucasian was 1.25 (95% CI, 1.01–1.54, P < 0.001). For RCC patients in low stage and under multivariate analyses, UCSI was also significant associated with poor CSS (P < 0.001 and P = 0.035, respectively), which indicated that UCSI might be an independent cancer-special outcome prognostic factor, especially in low stage RCC patients.

**Figure 3:**
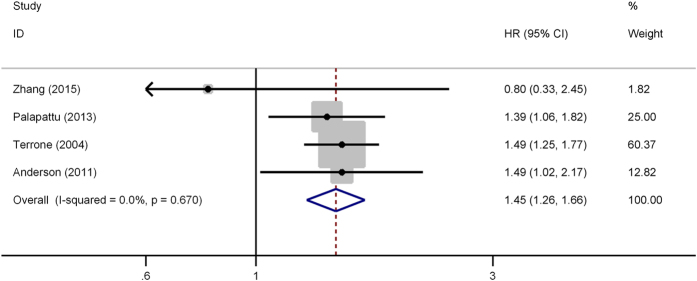
Forest plot of studies evaluating the association between urinary collecting system invasion and overall survival of renal cell carcinoma. HR = hazard ratio; CI = confidence interval.

No evident inter-study heterogeneity was observed in the 4 studies that focused on OS (*I*^*2*^ = 0%, P = 0.67). Thus, a fixed model was applied to pool the results. The combined HR for OS was 1.45 (95% CI, 1.26–1.66, P < 0.001), indicating that UCSI was associated with worse OS in patients with RCC (Fig. 3). In addition, sensitivity analysis was conducted by sequential omission of individual studies, which did not significantly influence the results and confirmed the credibility of outcomes.

**Figure 4:**
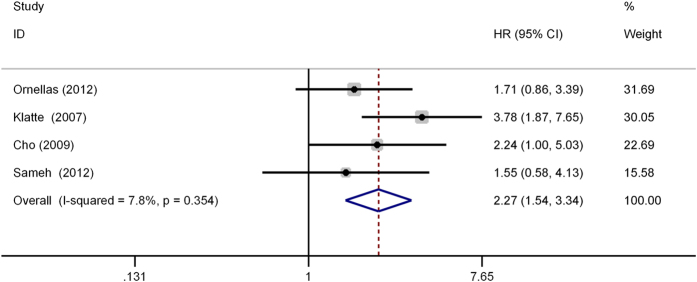
Forest plot of studies evaluating the association between urinary collecting system invasion and recurrence-free survival of renal cell carcinoma. HR = hazard ratio; CI = confidence interval.

As shown in Fig. 4, four studies were eligible for examining the relationship between the UCSI and RFS of RCC. A fixed effect model was selected because there was no evident heterogeneity among the four studies (*I*^*2*^ = 7.8%, P = 0.354). The pooled results (HR = 2.27, 95% CI: 1.54–3.34, P < 0.001) indicated that UCSI had an adverse impact on the RFS of RCC patients who received surgical treatment. The further sensitivity analysis did not alter the significance of combined HR, which validated the credibility of results.

### Publication bias

**Figure 5:**
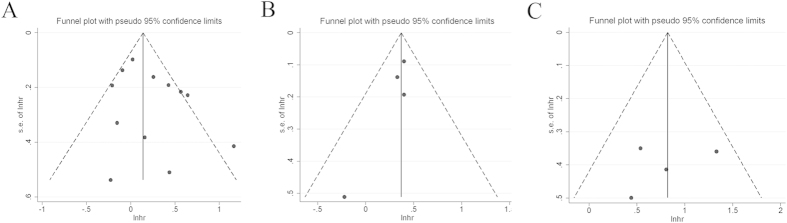
Funnel plots for the evaluation of potential publication bias. (**A**) cancer-specific survival; (**B**) overall survival; (**C**) recurrence-free survival.

Funnel plots, Begg’s test and Egger’s test were conducted to assess the publication bias in our meta-analysis of included studies. As shown in Fig. 5, there was no evident asymmetry in the funnel plots. In addition, the results from Begg’s test (P value) and Egger’s test (intercept with corresponding 95% CI, P value) for the included studies evaluating the survival outcomes were P_begg_ = 0.304, intercept 1.25 with 95% CI −0.86 to 3.36, P_egger_ = 0.216 (CSS); P_begg_ = 0.308, intercept −1.13 with 95% CI −3.31 to 1.04, P_egger_ = 0.154 (OS); P_begg_ = 1.000, intercept −2.76 with 95% CI −21.49 to 15.98, P_egger_ = 0.592 (RFS), respectively. Therefore, the aforementioned evidences suggested a low probability of publication bias.

## Discussion

The relationship between UCSI and oncological outcomes in RCC patients has attracted extensive attention and been widely debated, however, the reports remain controversial and there has yet to be a consensus on whether UCSI should be included in the following AJCC staging system^25^,^26^. Thus, we systemically review the published studies that evaluated the impact of UCSI on RCC survival and conducted a standard meta-analysis to clarify the prognostic value of UCSI in patients with RCC.

In the present research, based on the inclusion and quality assessment criteria, 17 studies were eligible and the HRs of cumulative survival rates were summarized quantitatively by meta-analysis techniques. Our results indicated that UCSI had a significant negative impact on OS and RFS of RCC patients who underwent surgically treatment. Interesting, by subgroup analysis, renal pelvis invasion was significantly associated with poor disease-specific prognosis in stage T1–T2 RCC (HR = 2.05, 95% CI: 1.43–2.96, P < 0.001) but not in stage T3–T4 tumors (HR = 1.08, 95% CI: 0.63–1.85, P = 0.771), which means that UCSI could predict cancer-specific mortality in organ-confined rather than in advanced tumors. The inconsistent prognostic influence suggests that pathological features such as perinephric fat involvement or vein invasion might be more important cancer-specific outcome predictors than UCSI in locally advanced tumors. On the other hand, it also suggests that collecting system invasion, together with tumor size, could be an additional useful prognostic variable for CSS in localized RCC.

To the best our knowledge, it is the first time that a comprehensive and standard meta-analysis has evaluated the association between UCSI and survival of kidney cancer. From our systematic review of 17 published studies including 9012 patients, the invasion of collecting system by RCC was unusual (11.2%), particularly in small masses. However, its prognostic value should not be ignored because the accurate determination of prognosis after surgery is highly important for both the planning of surveillance program and adequate adjuvant therapy. Our meta-analysis results quantity the impact of UCSI to be a negative prognostic factor. Several potential reasons for RCC patients with UCSI had a poor survival have been proposed but still not very clearly. A study by Klatte *et al*.^23^ showed that there was a tendency for an association between collecting system invasion and microvascular invasion and the biological aggressive of these tumors lead to poor survival. Besides, Waalkes *et al*.^10^ demonstrated that UCSI was significantly associated with increased frequencies of lymphatic and visceral metastasis at diagnosis, which indicated the invasion of renal pelvis might be a high risk factor for cancer recurrence.

There were also several limitations in our meta-analysis. First, although 17 eligible studies involving 9012 subjects were included in this systematic review, most of them were retrospective studies, which might render the results less reliable. Second, marked heterogeneity of studies was seen in pooled-analysis of CSS (*I*^2^ = 59.2%). By using subgroup analysis and meta-regression analysis, we found that the heterogeneity of CSS pooled-analysis may have been mostly due to different tumor stage among the included studies. When the analyses were performed separately according to low and high stage, the prognostic role of UCSI were significance and insignificance, respectively. Besides, several factors such as patients’ baseline characteristics (study size, gender, age, pathological subtype) and duration of follow up might also contribute to part of heterogeneity. Third, there were only four studies investigated OS and RFS of RCC even by a comprehensive literature search, which might inevitably increase the risk of random error, therefore more large prospective studies are needed to further confirm our findings. Finally, despite the well-recognized advantages of systematic review and meta-analysis, the results were affected by the quality of included studies and the reporting bias that papers with null or nonsignificant results were more difficult to be published than those with significant results might be unavoidable^27^.

Currently collecting system invasion is not considered as a criterion for tumor staging in the latest TNM staging system, thus some clinical pathologists fail to consistently describe this parameter in their reports even if present. In light of the absence of both centralized pathology and standardized system for classifying invasion, it might be argued that only extensive invasion into the collecting system was identified, while the less obvious or microscopic may have been missed, therefore leading to a selection bias of more aggressive tumors^28^. Further prospective evaluation of the relevance of collecting system invasion as a prognosticator is warranted and the complex prognostic implication of UCSI especially in organ-confined RCC might be one of the next challenges to be addressed by more high-quality studies in the future, which requires concise histopathological description on collecting system invasion to be rendered by pathological report.

In conclusion, our systematics review and meta-analysis of current evidence suggest that UCSI has a significant negative impact on OS and RFS in RCC patients and could be used to predict cancer-specific mortality in localized RCC. Thus, this pathological parameter might be recommended to consider for further TNM classification revisions and to improve the validly of new prognostic nomograms. Given the available data it does seem reasonable to conclude that RCC patients with collecting system invasion should be paid more attention by clinician and pathologist and require close follow up for their poor prognosis.

## Methods

### Literature search

This meta-analysis was conducted according to the guideline of Preferred Reporting Items for Systematic Reviews and Meta-Analyses (PRISMA)^29^. The detailed checklist of PRISMA 2009 was available in the supplementary data.

Electronic databases (PubMed, Embase, Web of Science and The Cochrane Library) were searched for published studies that investigated the relationship between UCSI and RCC prognosis up to June 2015. The search strategy included the following terms through MeSH headings, keywords, and text words: “collecting system invasion/involvement” or “renal pelvis invasion” or “pelvicaliceal invasion” combined with “renal cancer” or “renal cell carcinoma” or “kidney cancer”. Two independent investigators (Chen and Li) assessed the titles and abstracts of published studies. In addition, we manually reviewed the references cited in the relevant studies for possible inclusions. There was no language limitation existed in the search process.

### Eligibility criteria

The criteria for inclusion in our meta-analysis were set out as the following: (1) studies that confirmed the UCSI by histopathological examination; (2) studies analyzing the relationship between UCSI and RCC prognosis; (3) studies with the median follow-up not less than 12 months; (4) studies that reported overall survival (OS) or cancer-special survival (CSS) or recurrence-free survival (RFS) with hazard ratio (HR) and corresponding 95% confidence interval (CI) or studies that provided sufficient information to achieve an estimated HR and 95% CI by using the methods reported by Tierney *et al*.^30^.

Studies were excluded if they (1) were case reports, conference abstract, editorials or review articles; (2) investigated RCC cases fewer than 80 patients; (3) lacked sufficient data to estimate the HR and 95% CI. When multiple published papers by the same authors were retrieved, the most informative publication was included to avoid incorporating duplicated data.

Because the data included in our study were retrieved from published literature, ethical approval from ethics committees was not needed.

### Data extraction and quality assessment

Data from each eligible study were extracted by two investigators (Chen and Gu) independently with a standardized items form. The following information, if available, were recorded: first author’ name, publication year, study region, recruitment period, sample size, median of patient age, follow-up time and survival data including OS or CSS or RFS with their HRs and corresponding 95% CI.

Study quality was scored by two reviewers (Chen and Gu) using the Newcastle Ottawa Scale (NOS), which was recommended for the assessment of non-randomized studies^31^. The quality of studies included the following three main categories: selection, comparability and ascertainment of outcome. The total scores were added by these three aspects and a study with more scores means a better methodological quality. We defined studies with scores more than 6 were qualified to be included in the meta-analysis. Discrepancies between investigators for the above questions were resolved through discussion.

### Statistical analysis

Pooled HR with its corresponding 95% CI was calculated to evaluate the impact of UCSI on the survival of RCC patients, and HR greater than one indicated a worse prognosis in patients with UCSI. The statistical heterogeneity of combined HR was conducted using Cochrane Q test and *I*^*2*^ metrics. When there was no evident heterogeneity existed among studies (*I*^*2*^ > 50% suggested obvious heterogeneity)^32^, we used the fixed effect model, namely Mantel-haenszel method to pool the results, otherwise, the random effect model (DerSimonian and Laird method) was applied. Potential sources of heterogeneity, if significant, were explored by using subgroup analysis and meta-regression analysis. Besides, sensitivity analysis was performed by sequential omission of individual studies to evaluate the stability of outcomes. The possibility publication bias was assessed by visual inspection of the funnel plots, Begg rank correlation test^33^ and Egger linear regression test^34^. All analyses were performed using the program STATA version 12.0 (State Corporation, College Station, TX, USA). All statistical tests were two sided and difference was considered significant when a P value < 0.05.

## Additional Information

**How to cite this article**: Chen, L. *et al*. Prognostic role of urinary collecting system invasion in renal cell carcinoma: a systematic review and meta-analysis. *Sci. Rep.* **6**, 21325; doi: 10.1038/srep21325 (2016).

## Supplementary Material

Supplementary Information

## Figures and Tables

**Table 1:** Characteristics of eligible studies in the meta-analysis.

Author	Year	Country	Study design	Study period	Patient	UCSI	Age (median)	Follow up (median)	outcome	Quality scores
Zhang	2015	China	Cohort study	2008–2011	253 RCC	10	62.5	32.3 months	OS	8
Brookman-May	2011	Europe and USA	Cohort study	1984–2008	670 RCC (pT2)	75	59.4	51 months	CSS	8
Palapattu	2003	USA	Cohort study	1989–1999	895 RCC	124	59	31 months	OS	9
Klatte	2009	USA	Cohort study	1985–2007	158 pRCC	29	61.9	38 months	CSS	7
Margulis	2007	USA	Cohort study	1990–2006	365 RCC (pT3a)	34	58.2	22.5 months	CSS	8
Klatte	2007	USA	Cohort study	1985–2006	321 RCC (pT3)	112	60.9	28 months	CSS	7
Ornellas	2012	Brazil	Cohort study	2004–2010	227 RCC	38	60	28 months	RFS	8
Shi	2014	China	Cohort study	2000–2010	173 ccRCC	28	53	61.4 months	CSS	9
Klatte	2007	USA	Cohort study	1985–2005	519 RCC (pT1+pT2)	39	61	49 months	RFS	8
Cho	2009	Korea	Cohort study	1984–2007	299 ccRCC (pT1+pT2)	42	56	52.3 months	CSS, RFS	9
Terrone	2004	Italy	Cohort study	1983–1999	671 RCC	59	60.4	59 months	OS, CSS	9
Sameh	2012	Egypt	Cohort study	2000–2010	112 RCC (pT3+pT4)	10	59	24 months	RFS	7
Schrader	2009	Germany	Cohort study	1990–2005	780 RCC	67	64	5.44 years	CSS	8
Anderson	2011	USA	Cohort study	1988–2008	303 RCC (pT3)	67	61.8	23.3 months	OS, CSS	8
Verhoest	2009	France and Italy	Cohort study	1997–2004	754 RCC (pT1+pT2)	35	61	43 months	CSS	9
Waalkes	2010	Germany	Cohort study	1990–2005	1678 RCC	149	62	5.4 years	CSS	7
Brookman-Amissah	2010	Germany	Cohort study	1992–2006	834 RCC	90	62.2	79 months	CSS	9

Abbreviations: UCSI: urinary collecting system invasion; RCC: renal cell carcinoma; pRCC: papillary renal cell carcinoma; ccRCC: clear cell renal cell carcinoma; OS: overall survival; CSS: cancer-specific survival; RFS: recurrence-free survival.

**Table 2:** Meta-regression and subgroup analysis of the studies reporting the association of USCI and CSS of RCC.

Subgroup	Studies	Patients	Pooled HR	95% CI	Heterogeneity	Meta-regression *p*value
Ethnicity						0.820
Caucasian	10	6534	1.25	1.01–1.54	65.6%	
Asian	2	472	1.13	0.55–2.33	0%	
No. of patients						0.420
>500	6	5387	1.33	1.02–1.73	72.4%	
<500	6	1619	1.11	0.79–1.55	42.5%	
Stage						0.041
T_1–2_	3	1723	2.05	1.43–2.96	0%	
T_3–4_	3	989	1.08	0.63–1.85	74.5%	
Mixed	6	4294	1.10	0.94–1.30	26.7%	
Analysis						0.643
univariable analysis	2	664	1.02	0.59–1.75	0%	
multivariable analysis	10	6342	1.26	1.02–1.56	65.1%	

Abbreviations: UCSI: urinary collecting system invasion; CSS: cancer-specific survival; RCC: renal cell carcinoma; HR: hazard ratio; CI: confidence interval.

## References

[b1] DeSantis C. E. . Cancer treatment and survivorship statistics, 2014. CA Cancer J Clin 64, 252–271 (2014). 24890451 10.3322/caac.21235

[b2] Chow W. H., Dong L. M. & Devesa S. S. Epidemiology and risk factors for kidney cancer. Nat Rev Urol 7, 245–257 (2010). 20448658 10.1038/nrurol.2010.46 PMC3012455

[b3] Murai M. & Oya M. Renal cell carcinoma: etiology, incidence and epidemiology. Curr Opin Urol 14, 229–233 (2004). 15205579 10.1097/01.mou.0000135078.04721.f5

[b4] Patard J. J. . The changing evolution of renal tumours: a single center experience over a two-decade period. Eur Urol 45, 490–493 (2004). 15041114 10.1016/j.eururo.2003.12.015

[b5] Crispen P. L. . Lymph node dissection at the time of radical nephrectomy for high-risk clear cell renal cell carcinoma: indications and recommendations for surgical templates. Eur Urol 59, 18–23 (2011). 20933322 10.1016/j.eururo.2010.08.042

[b6] Ficarra V., Galfano A., Mancini M., Martignoni G. & Artibani W. TNM staging system for renal-cell carcinoma: current status and future perspectives. Lancet Oncol 8, 554–558 (2007). 17540307 10.1016/S1470-2045(07)70173-0

[b7] Carr D. T. The manual for the staging of cancer. Ann Intern Med 87, 491–492 (1977). 907249 10.7326/0003-4819-87-4-491

[b8] Anderson C. B. . Urinary collecting system invasion is a predictor for overall and disease-specific survival in locally invasive renal cell carcinoma. Urology 78, 99–104 (2011). 21550647 10.1016/j.urology.2011.02.039

[b9] Brookman-Amissah S. . Urinary collecting system invasion reflects adverse long-term outcome and is associated with simultaneous metastatic spread at the time of surgery and with multilocular dissemination during postsurgical follow-up in renal cell cancer. World J Urol 28, 103–109 (2010). 19479264 10.1007/s00345-009-0426-9

[b10] Waalkes S. . Urinary collecting system invasion is no independent prognostic factor in renal cell carcinoma. World J Urol 28, 283–288 (2010). 20237784 10.1007/s00345-010-0526-6

[b11] Schrader A. J. . Urinary collecting system invasion in renal cell carcinoma: incidence and long-term prognosis. Int J Urol 16, 718–722 (2009). 19659680 10.1111/j.1442-2042.2009.02353.x

[b12] Brookman-May S. . Collecting system invasion and Fuhrman grade but not tumor size facilitate prognostic stratification of patients with pT2 renal cell carcinoma. J Urol 186, 2175–2181 (2011). 22014800 10.1016/j.juro.2011.07.105

[b13] Klatte T. . Cytogenetic and molecular tumor profiling for type 1 and type 2 papillary renal cell carcinoma. Clin Cancer Res 15, 1162–1169 (2009). 19228721 10.1158/1078-0432.CCR-08-1229

[b14] Margulis V. . Location of extrarenal tumor extension does not impact survival of patients with pT3a renal cell carcinoma. J Urol 178, 1878–1882 (2007). 17868733 10.1016/j.juro.2007.07.011

[b15] Klatte T. . Prognostic factors for renal cell carcinoma with tumor thrombus extension. J Urol 178, 1189–1195 (2007). 17698087 10.1016/j.juro.2007.05.134

[b16] Shi X. . Prognostic prediction and diagnostic role of intercellular adhesion molecule-1 (ICAM1) expression in clear cell renal cell carcinoma. J Mol Histol 45, 427–434 (2014). 24535541 10.1007/s10735-014-9568-1

[b17] Cho H. J. . Prognostic value of capsular invasion for localized clear-cell renal cell carcinoma. Eur Urol 56, 1006–1012 (2009). 19135776 10.1016/j.eururo.2008.11.031

[b18] Terrone C. . Prognostic value of the involvement of the urinary collecting system in renal cell carcinoma. Eur Urol 46, 472–476 (2004). 15363563 10.1016/j.eururo.2004.07.006

[b19] Verhoest G. . Urinary collecting system invasion is an independent prognostic factor of organ confined renal cell carcinoma. J Urol 182, 854–859 (2009). 19616244 10.1016/j.juro.2009.05.017

[b20] yanhon Z., shejun M., shaofang W., zhiyong L. & ying W. Multivariate analysis of prognostic factors in renal cell carcinoma. Chinese Journal of Experimental Surgery 32, 638–640 (2015).

[b21] Palapattu G. S. . Collecting system invasion in renal cell carcinoma: impact on prognosis and future staging strategies. J Urol 170, 768–772 (2003). 12913694 10.1097/01.ju.0000082580.13597.a2

[b22] Ornellas A. A., Andrade D. M., Ornellas P., Wisnescky A. & de Santos Schwindt A. B. Prognostic factors in renal cell carcinoma: Analysis of 227 patients treated at the Brazilian national cancer institute. International Braz J Urol 38, 185–194 (2012). 22555027 10.1590/s1677-55382012000200006

[b23] Klatte T. . Prognostic relevance of capsular involvement and collecting system invasion in stage I and II renal cell carcinoma. BJU Int 99, 821–824 (2007). 17244281 10.1111/j.1464-410X.2006.06729.x

[b24] Sameh W. M., Hashad M. M., Eid A. A., Abou Yousif T. A. & Atta M. A. Recurrence pattern in patients with locally advanced renal cell carcinoma: The implications of clinicopathological variables. Arab Journal of Urology 10, 131–137 (2012). 26558015 10.1016/j.aju.2011.12.007 PMC4442897

[b25] Uzzo R. G., Cherullo E., Myles J. & Novick A. C. Renal cell carcinoma invading the urinary collecting system: implications for staging. J Urol 167, 2392–2396 (2002). 11992044

[b26] Moch H. . Reassessing the current UICC/AJCC TNM staging for renal cell carcinoma. Eur Urol 56, 636–643 (2009). 19595500 10.1016/j.eururo.2009.06.036

[b27] Sutton A. J., Song F., Gilbody S. M. & Abrams K. R. Modelling publication bias in meta-analysis: a review. Stat Methods Med Res 9, 421–445 (2000). 11191259 10.1177/096228020000900503

[b28] Lake A. M. & Chang S. S. Kidney cancer: The prognostic value of urinary collecting system invasion. Nat Rev Urol 6, 639–640 (2009). 19956192 10.1038/nrurol.2009.231

[b29] Moher D., Liberati A., Tetzlaff J., Altman D. G. & Group P. Preferred reporting items for systematic reviews and meta-analyses: the PRISMA statement. Int J Surg 8, 336–341 (2010). 20171303 10.1016/j.ijsu.2010.02.007

[b30] Tierney J. F., Stewart L. A., Ghersi D., Burdett S. & Sydes M. R. Practical methods for incorporating summary time-to-event data into meta-analysis. Trials 8, 16 (2007). 17555582 10.1186/1745-6215-8-16 PMC1920534

[b31] Wells G., Shea B., O’connell D., Peterson J. & Welch V. The Newcastle-Ottawa Scale (NOS) for assessing the quality of nonrandomised studies in meta-analyses. (2012) Available at: http://www.ohri.ca/programs/clinical_epidemiology/nosgen.pdf. (Accessed: 5 July 2015).

[b32] Higgins J. P., Thompson S. G., Deeks J. J. & Altman D. G. Measuring inconsistency in meta-analyses. BMJ 327, 557–560 (2003). 12958120 10.1136/bmj.327.7414.557 PMC192859

[b33] Begg C. B. & Mazumdar M. Operating characteristics of a rank correlation test for publication bias. Biometrics 50, 1088–1101 (1994). 7786990

[b34] Egger M., Davey Smith G., Schneider M. & Minder C. Bias in meta-analysis detected by a simple, graphical test. BMJ 315, 629–634 (1997). 9310563 10.1136/bmj.315.7109.629 PMC2127453

